# A randomized controlled trial of Baduanjin exercise to reduce the risk of atherosclerotic cardiovascular disease in patients with prediabetes

**DOI:** 10.1038/s41598-022-22896-5

**Published:** 2022-11-11

**Authors:** Xiaojun Ma, Manlin Li, Lin Liu, Fenfang Lei, Liduo Wang, Wenyan Xiao, Yingzi Tan, Binghua He, Sijie Ruan

**Affiliations:** 1 School of Nursing, Shaoyang University, Shaoyang, 422000 Hunan Province China; 2 Graduate School, Adamson University, 0900 Manila, Philippines; 3 School of Nursing, Yangzhou University, Yangzhou, 225009 Jiangsu Province China; 4 Department of Anesthesiology, Central Hospital of Shaoyang, Shaoyang City, 422000 Hunan Province China

**Keywords:** Diseases, Endocrinology, Health care, Medical research, Pathogenesis

## Abstract

To investigate the effectiveness of long-term Baduanjin and aerobic training on the 10-year risk of atherosclerotic cardiovascular disease in prediabetic patients. This study was single-blind randomized controlled trial. A total of 98 participants with prediabetes were randomly divided into three groups: the BDJ (n = 34), AT (n = 32), and control (n = 32) groups. Participants in the BDJ and AT groups underwent one year of supervised group exercise, consisting of 60 min/session every other day. The primary outcomes were metabolic control and the 10-year risk of ASCVD. The secondary outcome was a change in blood glucose status. After the intervention, various metabolic indexes were significantly improved in the two exercise groups relative to the control group and baseline measurements (*p* < 0.05). Compared with no exercise, BDJ and AT had significant preventive and protective effects against the risk of ASCVD in patients with prediabetes (*p* < 0.001). The overall effects of the two exercise groups were similar (*p* > 0.05). Long-term BDJ training can effectively reduce the risk of type 2 diabetes mellitus (T2DM) and its cardiovascular complications in prediabetic patients. The effect of BDJ is similar to that of moderate-intensity aerobic exercise.

## Introduction

Statistics released by the International Diabetes Federation (IDF)^1^ show that the incidence of type 2 diabetes mellitus (T2DM) has increased each year and that T2DM has become one of the largest epidemics of the twenty-first century. Prevention and control of T2DM are thus urgently needed. In 2021, 541 million individuals experienced impaired glucose tolerance worldwide; this number is predicted to increase to 730 million by 2045.The most important risk factor for T2DM is prediabetes^2^. According to a 20-year study on diabetes prevention conducted in Daqing, China^3^, 93% of patients with prediabetes will progress to T2DM within 20 years and therefore experience increased risk of cardiovascular disease (CVD).

Some studies have shown the risk of all-cause mortality (combined mortality due to CVD, coronary heart disease and stroke) is increased in people with prediabetes compared with those with normal blood glucose^4^. Compared with the Western population, prediabetic patients in the Asian population may progress to diabetes more quickly, and the risk of death is significantly higher among patients with atherosclerotic cardiovascular disease (ASCVD)^5,6^. Prediabetes is reversible; moreover, approximately 70% of CVD cases and deaths are attributed to modifiable risk factors^7^. Therefore, it is highly important to clinical practice to develop control strategies that further reduce blood glucose and ASCVD incidence in Asian patients with prediabetes.

Lifestyle interventions can effectively prevent diabetes^8,9^. The 30-year Daqing follow-up^10^ suggested that the risk of diabetes could be reduced through intensive lifestyle intervention, such as medical advice regarding nutrition and exercise; these changes also reduce the risk of cardiovascular complications, cardiovascular mortality and all-cause mortality among diabetic patients. Many studies^11–13^ have shown that moderate-intensity aerobic exercise, exercise at least three times a week, and exercising for ≥ 150 min per week not only ameliorates the abnormal glucose and lipid metabolism of patients with prediabetes but also controls hypertension, repairs damaged vascular endothelium, and reduces the risk of ASCVD. However, patients with prediabetes generally have more sedentary habits. These individuals are often restricted by factors such as old age, obesity, and spine and joint diseases, which make it difficult to keep up with the demands of aerobic exercise or remember the movements, thus dampening their enthusiasm for exercise^14^. Exercise motivation may also be affected by the surrounding environment and culture. Therefore, the Chinese guidelines for the prevention and treatment of diabetes recommend traditional exercises, such as Baduanjin, for patients to provide a greater variety of choices^2^. Baduanjin is one of the most widely practised fitness qigong in China and hasextensive links to traditional Chinese medicine and culture^15^. This type of exercise represents a nondrug treatment for diabetes prevention or management that stems from traditional Chinese medicine. Baduanjin emphasizes achieving balance of the body (i.e., posture) and mind as well as certain breathing skills during exercise; this practice is designed to open the meridians and activate the collaterals. These coordinated exercises draw from traditional Chinese medicine to strengthen the body and prevent disease. From a Western perspective, Baduanjin is categorized as low-intensity aerobic exercise and consists of 8 simple and slow movements that are easy to remember. Baduanjin exercise is therefore not limited by time or venue. Recent studies have shown that practising Baduanjin promotes the rehabilitation of patients with depression, sleep disorders, spinal and joint diseases and cognitive dysfunction^16,17^ and even improves the cardiopulmonary function of patients with heart failure^18^. Moreover, Baduanjin exercise plays a positive role in regulating the overall metabolic level in patients with T2DM. All these benefits indicate that engaging in Baduanjin may be an effective method to preventing and treating diabetes. However, research on this form of exercise has relatively short durations (average duration: 18 months)^19^, and controversy persists regarding the effects of practising Baduanjin on the disease outcomes of patients with prediabetes^20^. Unanswered questions in this field include the following. What advantage does Baduanjin have compared with other exercises? Is Baduanjin comparable to moderate-intensity aerobic exercise? Are the effects of practising Baduanjin equivalent to those of moderate-intensity aerobic exercise in reducing the incidence of diabetes and risk of ASCVD in patients with prediabetes? Convincing evidence from randomized controlled trials (RCTs) is lacking.

Therefore, in this research, an RCT was conducted to investigate changes in blood glucose, blood lipids, blood pressure, waist circumference (WC), and body weight in prediabetic patients after a year of Baduanjin training and to explore the effect of Baduanjin training on the 10-year risk of ASCVD in these patients. The aim of the RCT was to explore additional forms of exercise that facilitate the treatment of prediabetes and the prevention of cardiovascular complications and to broaden perspectives in the development of methods to promote physical and mental health.

## Results

### Participant demographics and clinical characteristics

**Figure 1:**
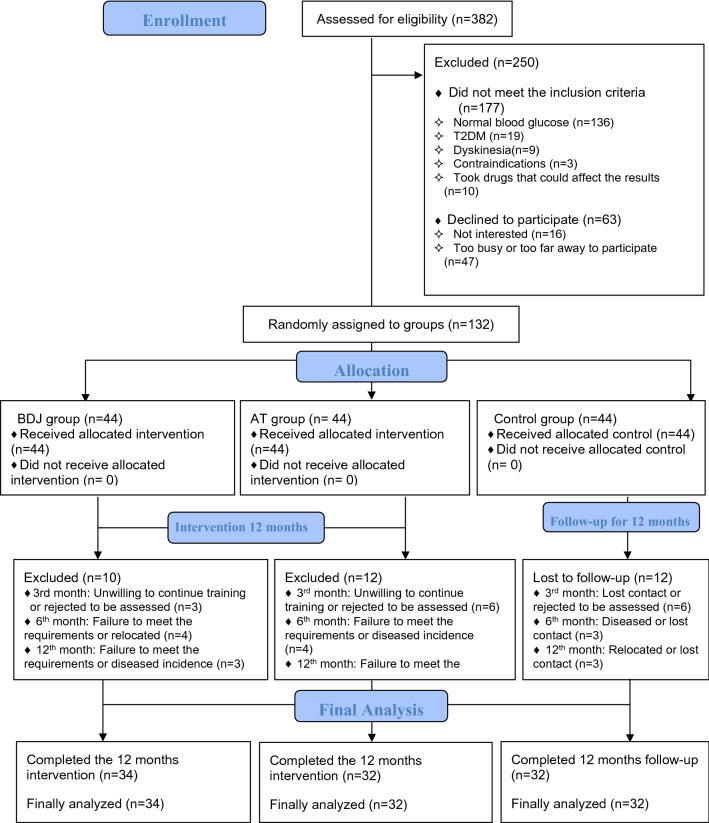
Flowchart of participant enrolment, allocation, and analysis.

As shown in Fig. 1, in May 2020, a total of 382 people were screened for this study, 132 of whom were eligible according to the inclusion criteria. A researcher used a random number table to randomly divide the participants into three groups, with 44 people in each group. After one year of intervention (from June 2020 to June 2021), 34 subjects were excluded due to relocation, disease incidence, substandard exercise, incomplete data or other factors. Thus, a total of 98 subjects completed this study, including 41 males and 57 females. Their average age was 59.35 ± 4.56 years. There were 34 participants in the BDJ group, 32 participants in the AT group and 32 participants in the control group. We also investigated the average attendance rate (%) of the two exercise groups, which was 88.24 ± 5.70% in the BDJ group and 87.29 ± 5.45% in the AT group. There was no significant difference in attendance rate between the two groups (t = 0.687, *p* = 0.495, d = 0.169).

### Baseline data

Table 1 shows the baseline data of the three groups. Chi-square tests and one-way analyses of variance (ANOVAs) revealed no significant group differences at baseline (*p* > 0.05), indicating that the baseline data on each dependent variable among the three groups were homogeneous. Among the participants in our study, 73(74.5%) were overweight or obese (BMI ≥ 24 kg/m^2^), 91(92.9%) exhibited abdominal obesity (WHtR > 0.5), 32(32.7%) were using antihypertensive drugs, and 31(31.6%) were currently smoking. Before the exercise intervention, some participants changed their dosage of antihypertensive drugs or smoking status, but during the intervention period, the numbers of participants who took antihypertensive drugs or smoked did not change; no participants took drugs that affected blood glucose or blood lipid profiles.

**Table 1:** Comparison of baseline demographic characteristics among groups [mean ± SD or n (%)].

Group index	Classification	Control (n = 32)	BDJ (n = 34)	AT(n = 32)	*F*/χ^2^	*p*
Height, m		1.58 ± 0.08	1.59 ± 0.09	1.58 ± 0.10	0.114	0.893
Age, years		59.09 ± 5.25	59.18 ± 3.93	59.81 ± 4.54	0.236	0.790
Sex	Male	14(43.8)	16(47.1)	11(34.4)	1.161	0.560
	Female	18(56.2)	18(52.9)	21(65.6)		
Use antihypertensive drugs	Yes	10(31.2)	10(29.4)	12(37.5)	0.533	0.766
	No	22(68.8)	24(70.6)	20(62.5)		
Currently smoke	Yes	10(31.2)	13(38.2)	8(25.0)	1.338	0.512
	No	22(68.8)	21(61.8)	24(75.0)		
Family history of ASCVD	Yes	27(84.4)	30(88.2)	29(90.6)	0.593	0.743
	No	5(15.6)	4(11.8)	3(9.4)		
Education	< 12 years	21(65.6)	25(73.5)	19(59.4)	1.489	0.475
	≥ 12 years	11(34.4)	9(26.5)	13(40.6)		
WHtR, %	≤ 0.5	1(3.1)	3(8.8)	3(9.4)	1.329	0.515
	> 0.5	31(96.9)	31(91.2)	29(90.6)		
BMI, kg/m^2^	< 24	7(21.9)	10(29.4)	8(25.0)	4.577	0.334
	24 to < 28	15(46.9)	19(55.9)	20(62.5)		
	≥ 28	10(31.2)	5(14.7)	4(12.5)		
FPG, mmol/L		6.13 ± 0.64	5.97 ± 0.56	5.91 ± 0.68	1.093	0.339
2-hPG, mmol/L		8.67 ± 0.95	8.46 ± 1.08	8.45 ± 1.36	0.374	0.689
HbA1c, %		6.11 ± 0.51	5.97 ± 0.40	5.87 ± 0.61	1.773	0.175
TC, mmol/L		5.34 ± 0.96	5.18 ± 0.80	5.22 ± 1.06	0.269	0.765
TG, mmol/L		2.20 ± 0.58	2.27 ± 0.60	2.18 ± 0.71	0.204	0.816
HDL-C, mmol/L		1.37 ± 0.48	1.36 ± 0.32	1.30 ± 0.26	0.331	0.719
LDL-C, mmol/L		3.56 ± 0.64	3.37 ± 0.74	3.49 ± 1.00	0.467	0.628
WC, cm		90.19 ± 5.97	88.62 ± 7.98	87.97 ± 7.91	0.770	0.466
Weight, kg		67.41 ± 11.98	64.71 ± 12.11	63.63 ± 12.52	0.817	0.445
SBP, mmHg		144.22 ± 14.35	145.65 ± 14.24	143.81 ± 14.05	0.153	0.859
DBP, mmHg		81.56 ± 8.71	80.88 ± 9.70	82.41 ± 8.12	0.243	0.785
10-year risks of ASCVD, %		6.13 ± 1.83	6.17 ± 3.07	6.06 ± 2.38	0.017	0.983

BDJ, Baduanjin; AT, aerobic training; FPG, fasting plasma glucose; 2-hPG, 2-h plasma glucose; HbA1c, haemoglobin A1c; TC, total cholesterol; TG, triglycerides; HDL-C, high-density lipoprotein cholesterol; LDL-C, low-density lipoprotein cholesterol; WC, waist circumference; SBP, systolic blood pressure; DBP, diastolic blood pressure; ASCVD, atherosclerotic cardiovascular disease; WHtR, waist-to-height ratio; BMI, body mass index.

### Primary outcomes

#### Comparison of blood glucose, blood lipid profiles, body shape, blood pressure and 10-year risk of ASCVD among the three groups

Tables 2, 3, 4, 5 report the results of two-factor repeated-measures ANOVAs used to assess group differences in the dependent variables. According to the Shapiro‒Wilk test, all dependent variables were essentially normally distributed (*p* > 0.05). Box’s M test indicated that the variance–covariance matrix of the dependent variables were equal (*p* > 0.001). According to Mauchly’s sphericity test, none of the dependent variables met the assumption of sphericity (*p* < 0.05). Therefore, the interaction effects, main effects, simple effects and results of the post hoc multiple comparisons are subject to the results of the multivariate ANOVA. Levene’s test indicated that the variances of the dependent variables were equal (*p* > 0.05). The repeated-measures ANOVAs indicated a significant effect of the Group × Time interaction on each dependent variable (*p* < 0.05); therefore, dependent variables in each group exhibited different changes over time. There were significant main effect of the Group and Time on each dependent variable (*p* < 0.05).

**Table 2:** Effects of interventions on blood glucose indicators among the three groups [mean ± SD or mean difference (95% CI)].

Index and time point	Group			Cohen's d			Repeated-measures ANOVA		
	Control(n = 32)	BDJ (n = 34)	AT (n = 32)	BDJ versus Control	AT versus Control	BDJ versus AT	Group × Time	Time	Group
**FPG, mmol/L**									
T0	6.13 ± 0.64	5.97 ± 0.56	5.91 ± 0.68	− 0.27	− 0.33	0.10			
T3	6.24 ± 0.41	5.83 ± 0.46	5.71 ± 0.62	− 0.94#	− 1.01#	0.22			
T6	6.35 ± 0.37	5.63 ± 0.51	5.51 ± 0.61	− 1.61#	− 1.67#	0.21			
T12	6.49 ± 0.56	5.56 ± 0.66	5.41 ± 0.60	− 1.52#	− 1.86#	0.24			
T3 versus T0	0.11(− 0.03, 0.25)	− 0.14(− 0.28, − 0.001)*	− 0.20(− 0.35, − 0.6)*			F	15.860	4.546	17.798
T6 versus T0	0.22(0.040, 0.4)*	− 0.34(− 0.51, − 0.17)#	− 0.40(− 0.58, − 0.22)#			*p*	0.001	0.005	0.001
T12 versus T0	0.36(0.16, 0.55)#	− 0.41(− 0.60, − 0.22)#	− 0.50(− 0.69, − 0.30)#			η^2^p	0.336	0.128	0.273
**2-hPG,mmol/L**									
T0	8.67 ± 0.95	8.46 ± 1.08	8.45 ± 1.36	− 0.21	− 0.19	0.01			
T3	8.76 ± 0.97	8.12 ± 0.92	8.17 ± 0.99	− 0.68*	− 0.61*	− 0.04			
T6	8.90 ± 1.09	7.87 ± 1.07	7.63 ± 0.93	− 0.95#	− 1.25#	0.24			
T12	9.15 ± 1.08	7.70 ± 1.23	7.68 ± 0.88	− 1.25#	− 1.49#	0.02			
T3 versus T0	0.10(− 0.20, 0.40)	− 0.34(− 0.63, − 0.05)*	− 0.28(− 0.58, 0.02)			F	11.075	7.109	10.172
T6 versus T0	0.23(− 0.08, 0.55)	− 0.58(− 0.89, − 0.28)#	− 0.81(− 1.13, − 0.50)#			*p*	< 0.001	0.001	< 0.001
T12 versus T0	0.48(0.10, 0.87)*	− 0.76(− 1.14, − 0.39)#	− 0.77(− 1.16, − 0.38)#			η2p	0.261	0.165	0.176
**HbA1c, %**									
T0	6.11 ± 0.51	5.97 ± 0.40	5.87 ± 0.61	− 0.31	− 0.43	0.20			
T3	6.14 ± 0.45	5.93 ± 0.38	5.66 ± 0.63	− 0.51	− 0.88#	0.52*			
T6	6.24 ± 0.49	5.79 ± 0.37	5.59 ± 0.53	− 1.04#	− 1.27#	0.44			
T12	6.29 ± 0.47 ^b^	5.70 ± 0.42	5.43 ± 0.61	− 1.33#	− 1.58#	0.52*			
T3 versus T0	0.03(− 0.10, 0.16)	− 0.04(− 0.17, 0.08)	− 0.21(− 0.34, − 0.08)*			F	13.392	5.718	13.185
T6 versus T0	0.14(− 0.02, 0.29)	− 0.18(− 0.32, − 0.03)*	− 0.28(− 0.43, − 0.13)#			*p*	< 0.001	0.001	< 0.001
T12 versus T0	0.18(− 0.03, 0.34)*	− 0.27(− 0.42, − 0.12)#	− 0.43(− 0.59, − 0.28)#			η^2^p	0.299	0.156	0.217

BDJ, Baduanjin; AT, aerobic training; FPG, fasting plasma glucose; 2-hPG, 2-h plasma glucose; HbA1c, haemoglobin A1c; η^2^p, partial eta-squared; T0, baseline; T3, at 3 months; T6, at 6 months; T12, at 12 months; *, significant difference at *p* < 0.05; #, significant difference at *p* < 0.001.

**Table 3:** Effects of interventions on blood lipid profiles among the three groups [mean ± SD or mean difference (95% CI)].

Index and time point	Group			Cohen's d			Repeated-measures ANOVA		
	Control(n = 32)	BDJ (n = 34)	AT (n = 32)	BDJ versus Control	AT versus Control	BDJ versus AT	Group × Time	Time	Group
**TC, mmol/L**									
T0	5.34 ± 0.96	5.18 ± 0.80	5.22 ± 1.06	− 0.18	− 0.12	− 0.04			
T3	5.37 ± 0.80	5.01 ± 0.86	5.00 ± 0.99	− 0.43	− 0.41	0.01			
T6	5.38 ± 0.82	4.91 ± 0.93	4.79 ± 0.83	− 0.54*	− 0.72*	0.14			
T12	5.49 ± 0.89	4.74 ± 0.92	4.66 ± 0.93	− 0.83#	− 0.91#	0.09			
T3 versus T0	0.02(− 0.15 , 0.20)	− 0.16(− 0.34 , 0.01)	− 0.21(− 0.39, − 0.04)*			F	5.917	5.239	3.318
T6 versus T0	0.04(− 0.17 , 0.25)	− 0.27(− 0.48 , − 0.07)*	− 0.43(− 0.65, − 0.22)#			*p*	0.001	0.002	0.040
T12 versus T0	0.15(− 0.10 , 0.41)	− 0.44(− 0.69 , − 0.19)#	− 0.56(− 0.82 − 0.31)#			η^2^p	0.159	0.145	0.065
**TG, mmol/L**									
T0	2.20 ± 0.58	2.27 ± 0.60	2.18 ± 0.71	0.12	− 0.03	0.14			
T3	2.24 ± 0.62	2.10 ± 0.61	1.93 ± 0.69	− 0.23	− 0.47	0.26			
T6	2.38 ± 0.67	2.03 ± 0.65	1.81 ± 0.73	− 0.53*	− 0.81#	0.32			
T12	2.48 ± 0.64	1.91 ± 0.69	1.76 ± 0.64	− 0.86#	− 1.13#	0.23			
T3 versus T0	0.04(− 0.07, 0.14)	− 0.17(− 0.27, − 0.07)*	− 0.25(− 0.35, − 0.14)#			F	25.138	8.406	3.463
T6 versus T0	0.18(0.03, 0.32)*	− 0.24(− 0.39, − 0.10)#	− 0.36(− 0.51, − 0.22)#			*p*	0.001	0.001	0.035
T12 versus T0	0.28(0.15, 0.41)#	− 0.36(− 0.48, − 0.24)#	− 0.41(− 0.54, − 0.29)#			η^2^p	0.445	0.213	0.068
**HDL-C, mmol/L**									
T0	1.37 ± 0.48	1.36 ± 0.32	1.30 ± 0.26	− 0.02	− 0.18	0.21			
T3	1.37 ± 0.26	1.46 ± 0.29	1.45 ± 0.33	0.33	0.27	0.03			
T6	1.39 ± 0.25	1.59 ± 0.36	1.58 ± 0.25	0.64*	0.76#	0.03			
T12	1.29 ± 0.25	1.98 ± 0.36	1.72 ± 0.23	2.21#	1.79#	0.86#			
T3 versus T0	0.003(− 0.11, 0.12)	0.11(− 0.01, 0.22)	0.15(0.04, 0.27)*			F	25.183	27.511	9.025
T6 versus T0	0.02(− 0.11, 0.15)	0.24(0.11, 0.36)#	0.28(0.151, 0.4)#			*p*	< 0.001	< 0.001	< 0.001
T12 versus T0	− 0.08(− 0.21, 0.05)	0.63(0.50, 0.75)#	0.43(0.30, 0.55)#			η^2^p	0.446	0.470	0.160
**LDL-C, mmol/L**									
T0	3.56 ± 0.64	3.37 ± 0.74	3.49 ± 1.00	− 0.27	− 0.08	− 0.14			
T3	3.49 ± 0.67	3.18 ± 0.88	3.36 ± 0.93	− 0.39	− 0.16	− 0.20			
T6	3.55 ± 0.68	3.01 ± 0.95	3.12 ± 0.78	− 0.66*	− 0.59*	− 0.14			
T12	3.77 ± 0.75	2.91 ± 0.83	2.92 ± 0.93	− 1.09#	− 1.01#	− 0.01			
T3 versus T0	− 0.06(− 0.21, 0.08)	− 0.19(0.33, 0.05)*	− 0.13(− 0.27, 0.02)			F	7.630	7.913	3.627
T6 versus T0	− 0.01(− 0.19, 0.16)	− 0.36(− 0.53, − 0.19)#	− 0.37(− 0.54, − 0.19)#			*p*	< 0.001	< 0.001	0.030
T12 versus T0	0.21(− 0.05, 0.47)	− 0.46(− 0.71, − 0.21)#	− 0.56(− 0.82, − 0.31)#			η^2^p	0.196	0.203	0.071

BDJ, Baduanjin; AT, aerobic training; TC, total cholesterol; TG, triglycerides; HDL-C, high-density lipoprotein cholesterol; LDL-C, low-density lipoprotein cholesterol; η^2^p, partial eta-squared; T0, baseline; T3, at 3 months; T6, at 6 months; T12, at 12 months; *, significant difference at *p* < 0.05; #, significant difference at *p* < 0.001.

**Table 4:** Effects of interventions on body shape indicators among the three groups [mean ± SD or mean difference (95% CI)].

Index and time point	Group			Cohen's d			Repeated-measures ANOVA		
	control(n = 32)	BDJ (n = 34)	AT (n = 32)	BDJ versus Control	AT versus Control	BDJ versus AT	Group × Time	Time	Group
**WC, cm**									
T0	90.19 ± 5.97	88.62 ± 7.98	87.97 ± 7.91	− 0.22	− 0.32	0.08			
T3	90.25 ± 6.00	86.82 ± 7.59	85.84 ± 7.59	− 0.50	− 0.64*	0.13			
T6	92.94 ± 5.29	84.71 ± 7.30	83.38 ± 7.09	− 1.28#	− 1.53#	0.18			
T12	93.25 ± 5.59	82.41 ± 7.07	79.88 ± 6.68	− 1.69#	− 2.17#	0.37			
T3 versus T0	0.06(− 0.75 0.87)	− 1.79(− 2.58, − 1.01)#	− 2.13(− 2.93, − 1.32)#			F	69.229	45.508	11.098
T6 versus T0	2.75(1.70, 3.80)#	− 3.91(− 4.93, − 2.89)#	− 4.59(− 5.65, − 3.54)#			*p*	< 0.001	< 0.001	< 0.001
T12 versus T0	3.06(1.89, 4.24)#	− 6.21(− 7.34, − 5.07)#	− 8.09(− 9.27, − 6.92)#			η^2^p	0.688	0.595	0.189
**Weight, kg**									
T0	67.41 ± 11.98	64.71 ± 12.11	63.63 ± 12.52	− 0.22	− 0.31	0.09			
T3	68.16 ± 10.79	62.85 ± 12.11	61.63 ± 11.82	− 0.46	− 0.58	0.10			
T6	69.31 ± 10.78	61.82 ± 12.19	60.66 ± 12.21	− 0.65*	− 0.75*	0.10			
T12	69.84 ± 10.12	60.41 ± 11.26	58.66 ± 11.98	− 0.88#	− 1.01#	0.15			
T3 versus T0	0.75(− 0.11, 1.61)	− 1.85(− 2.68, − 1.02)#	− 2.00(− 2.86, − 1.14)#			F	23.863	10.475	3.925
T6 versus T0	1.91(0.86, 2.96)#	− 2.88(− 3.90, − 1.86)#	− 2.97(− 4.02, − 1.92)#			P	< 0.001	< 0.001	0.023
T12 versus T0	2.44(1.01, 3.87)#	− 4.29(− 5.68, − 2.91)#	− 4.97(− 6.40, − 3.54)#			η^2^p	0.432	0.253	0.076

Abbreviations: BDJ, Baduanjin; AT, aerobic training; WC, waist circumference; η^2^p, partial eta-squared; T0, baseline; T3, at 3 months; T6, at 6 months; T12, at 12 months; *, significant difference at *p* < 0.05; #, significant difference at *p* < 0.001.

**Table 5:** Effects of interventions on BP and 10-year risks of ASCVD among the three groups [mean ± SD or mean difference (95% CI)].

Index and time point	Group			Cohen's d			Repeated-measures ANOVA		
	control(n = 32)	BDJ (n = 34)	AT (n = 32)	BDJ versus Control	AT versus Control	BDJ versus AT	Group × Time	Time	Group
**SBP, mmHg**									
T0	144.22 ± 14.35	145.65 ± 14.24	143.81 ± 14.05	0.10	− 0.03	0.13			
T3	143.31 ± 13.93	137.97 ± 12.32	134.53 ± 10.62	− 0.41	− 0.71*	0.30			
T6	145.31 ± 15.13	136.38 ± 13.51	133.44 ± 10.58	− 0.62*	− 0.91#	0.24			
T12	146.50 ± 14.28	136.59 ± 12.13	131.94 ± 11.53	− 0.75*	− 1.12#	0.39			
T3 versus T0	− 0.91(− 3.25, 1.44)	− 7.68(− 9.95, − 5.40)#	− 9.28(− 11.62, − 6.94)#			F	31.605	33.403	4.067
T6 versus T0	1.09(− 1.41, 3.59)	− 9.27(− 11.69, − 6.84)#	− 10.38(− 12.88,− 7.88)#			*p*	< 0.001	< 0.001	0.020
T12 versus T0	2.28(0.07, 4.49)*	− 9.06(− 11.21,− 6.91)#	− 11.88(− 14.09,− 9.66)#			η^2^p	0.502	0.519	0.079
**DBP, mmHg**									
T0	81.56 ± 8.71	80.88 ± 9.70	82.41 ± 8.12	− 0.07	0.10	− 0.17			
T3	83.16 ± 8.22	78.21 ± 9.10	79.28 ± 9.22	− 0.57*	− 0.44	− 0.14			
T6	84.59 ± 7.49	78.03 ± 8.10	76.31 ± 8.11	− 0.84#	− 1.06#	0.21			
T12	85.63 ± 8.04	77.35 ± 8.92	75.41 ± 7.62	− 0.97#	− 1.30#	0.23			
T3 versus T0	1.59(0.15, 3.04)*	− 2.68(− 4.08, − 1.27)#	− 3.13(− 4.57, − 1.68)#			F	38.978	9.925	4.611
T6 versus T0	3.03(1.52, 4.54)#	− 2.85(− 4.32, − 1.39)#	− 6.09(− 7.61, − 4.58)#			*p*	< 0.001	< 0.001	0.012
T12 versus T0	4.06(2.51, 5.62)#	− 3.53(− 5.04, − 2.02)#	− 7.00(− 8.56, − 5.44)#			η^2^p	0.554	0.243	0.088
**10-year risks of ASCVD, %**									
T0	6.13 ± 1.83	6.17 ± 3.07	6.06 ± 2.38	0.02	− 0.03	0.04			
T3	5.96 ± 1.68	5.03 ± 1.98	4.83 ± 1.74	− 0.51*	− 0.66*	0.11			
T6	6.25 ± 2.07	4.66 ± 1.84	4.48 ± 1.64	− 0.81#	− 0.95#	0.10			
T12	7.08 ± 2.86	4.12 ± 1.76	4.03 ± 1.50	− 1.26#	− 1.34#	0.05			
T3 versus T0	− 0.18(− 0.62, 0.27)	− 1.14(− 1.57, − 0.71)#	− 1.23(− 1.68, − 0.79)#			F	22.238	15.490	6.002
T6 versus T0	0.12(− 0.41, 0.64)	− 1.51(− 2.02, − 0.10)#	− 1.58(− 2.11, − 1.05)#			*p*	< 0.001	< 0.001	0.004
T12 versus T0	0.95(− 0.33, − 1.57)*	− 2.05(− 2.66, − 1.45)#	− 2.03(− 2.65, − 1.41)#			η^2^p	0.415	0.333	0.112

BDJ, Baduanjin; AT, aerobic training; SBP, systolic blood pressure; DBP, diastolic blood pressure; ASCVD, atherosclerotic cardiovascular disease; η^2^p, partial eta-squared; T0, baseline; T3, at 3 months; T6, at 6 months; T12, at 12 months; *, significant difference at *p* < 0.05; #, significant difference at *p* < 0.001.

As shown in Table 2, at the 3rd month of intervention, the two exercise groups exhibited significant decreases in FPG and 2-hPG compared to the control group (*p* < 0.05), and the improvement effect was more obvious over time. Additionally, the AT group exhibited significant decreases in HbA1c (d = − 0.88, *p* < 0.001) compared to the control group. The same improvement in HbA1c was observed in the BDJ group at the 6th month of intervention. Notably, there was a significant difference in HbA1c between the BDJ group and AT group at the 3rd month (d = 0.52, *p* = 0.029) and 12th month (d = 0.52, *p* = 0.036) of the intervention; aerobic exercise improved HbA1c to a greater extent. Compared with the baseline, the blood glucose and HbA1c in the control group increased significantly from the 6 to 12 months of follow-up (*p* < 0.05), while that in the two exercise groups decreased significantly from the 3rd month of intervention (*p* < 0.05).

As shown in Table 3, at the 6th month of intervention, the BDJ and AT groups exhibited significant decreases in TC, TG and LDL-C as well as significant increases in HDL-C compared to the control group (*p* < 0.05). At the 12th month of intervention, compared with the baseline, the control group had no significant changes in other indicators except for a significant increase in TG (*p* < 0.05), while the dyslipidemia of the two exercise groups was significantly improved (*p* < 0.05). Notably, At the 12th month of intervention, HDL-C in the BDJ group was significantly higher than that in the AT group(d = 0.86, *p* < 0.001) , and the HDL-C increase in the BDJ group [0.63 (0.50, 0.75)] was greater than that in the AT group [0.43 (0.30, 0.55)], and HDL-C increased by 46.32% and 33.08%, respectively; this difference was significant (d = 0.68, *p* = 0.027).

Table 4 shows the comparison of body shape indicators. From the 6th month of intervention, the BDJ and AT groups exhibited significant decreases in WC and Weight compared to the control group (*p* < 0.05). Compared with the baseline, the WC and Weight of the control group increased significantly from the 6th month of follow-up (*p* < 0.05), while that in the two exercise groups decreased significantly from the 3rd month of intervention (*p* < 0.001). At the 12th month of intervention, the weight of participants in the BDJ and AT groups decreased by 6.63% and 7.81%, respectively, and their WC decreased by 7% and 9.2%.

As shown in Table 5, in the control group, at the 12-month follow-up, the 10-year risk of ASCVD in the control group was significantly higher than that at baseline [0.95 (− 0.33, − 1.57), *p* = 0.003]; the risk level increased by 15.50%. Compared with the control group, at the 3rd month of intervention, the two exercise groups exhibited significant decreases in the 10-year risk of ASCVD (BDJ: d = − 0.51, *p* = 0.040; AT: d = − 0.66, *p* = 0.014). Over the intervention period, the improvement persisted and became more obvious with intervention duration. By the 12th month of intervention, the 10-year risk of ASCVD in the BDJ group and AT group was significantly lower than that at baseline [BDJ: − 2.05 (− 2.66, − 1.45), *p* < 0.001; AT: − 2.03 (− 2.65, − 1.41)], *p* < 0.001], and the risk level was reduced by 33.23% and 33.50%, respectively. Additionally, the SBP of participants in the BDJ and AT groups decreased by 6.22% and 8.26%. The decrease in DBP in the BDJ group [− 3.53 (− 5.04, − 2.02)] was smaller than that in the AT group [− 7.00 (− 8.56, − 5.44)], and DBP decreased by 4.36% and 8.49%, respectively; this difference was significant (d = 0.382, *p* = 0.002).

#### Cox proportional hazards model for 10-year ASCVD risk

**Figure 2:**
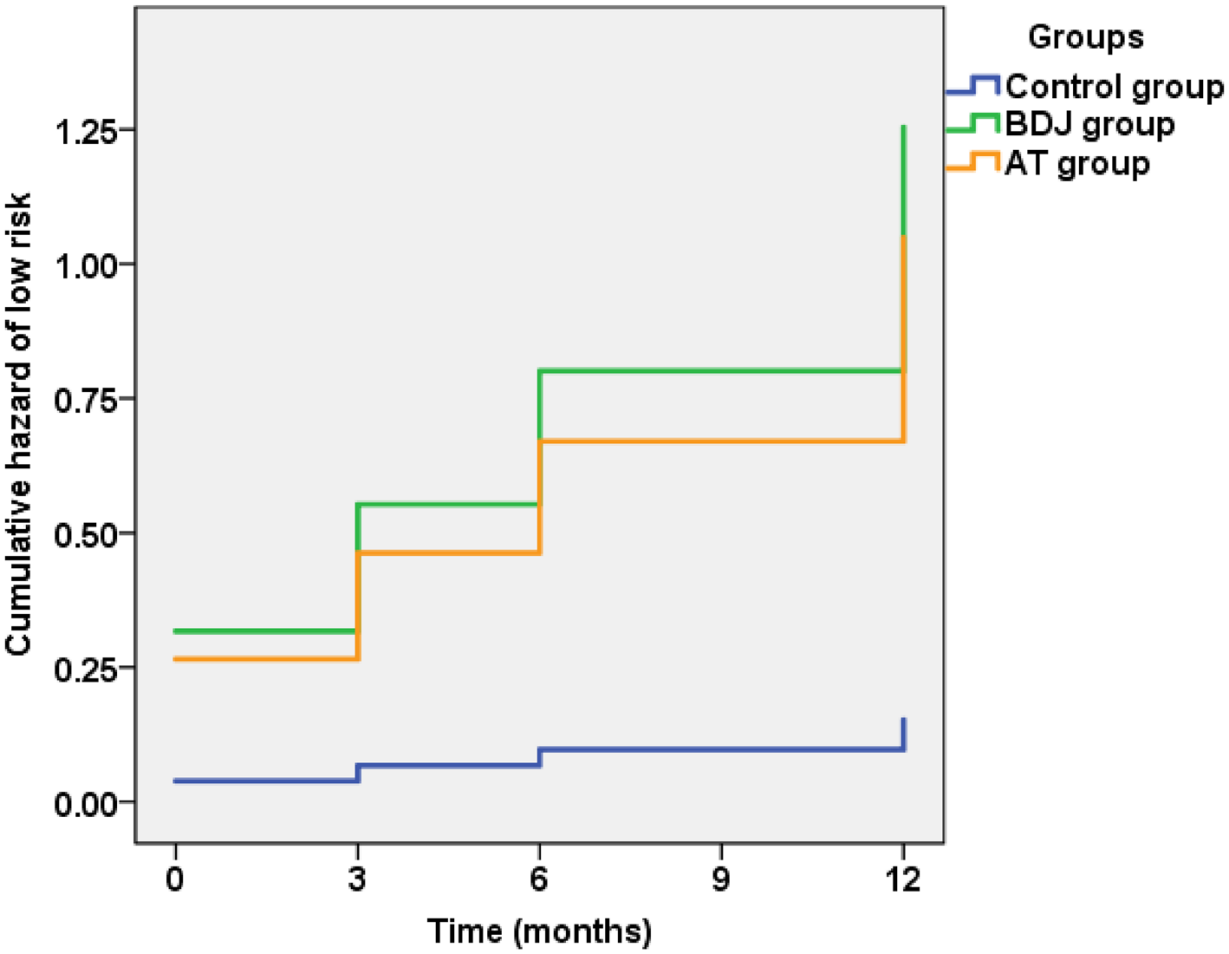
The change trend of 10-year ASCVD risk in the 3 groups.

Table 6 and Fig. 2 show the results of the Cox proportional hazards model. Among the 98 participants, 55 (56.1%) participants exhibited a decrease in their 10-year risk of ASCVD, changing to low risk, and 43 participants exhibited risk levels that remained at or changed to medium or high risk. The results of the omnibus test show the significance of the new model (*p* < 0.001). Among the included variables, the exercise intervention modes, age and use of antihypertensive drugs were important influencing factors for the 10-year risk of ASCVD in patients with prediabetes. Additionally, compared with the control group, the 10-year risk of ASCVD in the BDJ group [hazard ratio (HR) = 8.242, 95% confidence interval (CI):3.213 to 21.141, *p* < 0.001] and AT group [HR = 6.895, 95% CI (2.693 to 17.654), *p* < 0.001] decreased significantly. In addition, for patients with hypertension, use of antihypertensive drugs reduced the 10-yearrisk of ASCVD [HR = 2.758, 95% CI (1.379 to 5.514), *p* = 0.004].

**Table 6:** Cox proportional hazards model analysis of changes in the 10-year ASCVD risk.

Factor	HR	95% CI	*p*
BDJ	8.242	3.213 to 21.141	< 0.001
AT	6.895	2.693 to 17.654	< 0.001
Height	1.019	0.004 to 263.999	0.995
Age	0.896	0.841 to 0.956	< 0.001
Sex	3.005	0.654 to 13.811	0.157
Current smoker	0.883	0.246 to 3.168	0.849
Use of antihypertensive drugs	2.758	1.379 to 5.514	0.004
Family history of ASCVD	1.660	0.765 to 3.601	0.200

BDJ, Baduanjin; AT, aerobic training; ASCVD, atherosclerotic cardiovascular disease.

### Secondary outcomes

As shown in Table 7, prediabetes was divided into IFG and IGT. Before the intervention, there was no significant difference in the number of participants with IFG or IGT among the three groups (χ^2^ = 2.541, *p* = 0.281). However, by the 12th month of intervention, there were significant differences in the number of people with different blood glucose statuses among the three groups (χ^2^ = 47.99, *p* < 0.001). Compared with the control group, there were significant increases in the number of people whose blood glucose status improved in the BDJ group (χ^2^ = 42.533, *p* < 0.001, d = 2.693) and AT group (χ^2^ = 23.259, *p* < 0.001, d = 1.511). Although the number of people whose blood glucose changed to normal in the BDJ group (67.6%) was higher than that in the AT group (53.1%), this difference was not significant (χ ^2^ = 3.236, *p* = 0.198, d = 0.454).

**Table 7:** Change in blood glucose metabolism.

Group	Blood glucose status	Change in blood glucose metabolism		
		Transition to NGR	Remained prediabetic	Transition to T2DM
Control (n = 32)	IFG(n = 6)	0	6	0
	IGT(n = 26)	0	23	3
	Sum(n,%)	0(0.0)	29(90.6)	3(9.4)
BDJ (n = 34)	IFG(n = 7)	6	1	0
	IGT(n = 27)	17	9	1
	Sum(n,%)	23(67.6)	10(29.4)	1(2.9)
AT (n = 32)	IFG(n = 11)	6	5	0
	IGT(n = 21)	11	10	0
	Sum(n,%)	17(53.1)	15(46.9)	0(0.0)

BDJ, Baduanjin; AT, aerobic training; IFG, impaired fasting glucose; IGT, impaired glucose tolerance; NGR, normal glucose regulation; T2DM,type 2 diabetes mellitus.

### Safety outcomes

We conducted a safety assessment when screening participants for exercise capabilities, and those who did not meet the inclusion criteria were excluded. Two weeks before the intervention, participants were provided with adaptive training to adjust to the exercise intensity. During the intervention, we monitored the subjective feelings, BP and heart rate of participants. Those who could not continue due to disease or other reasons withdrew. No adverse events occurred during the intervention.

## Discussion

The results of this study show that long-term (a year) engagement in Baduanjin and aerobic exercise improved the blood glucose, blood lipid profile, BP and body shape of patients with prediabetes and significantly reduced the risk of diabetes and the 10-year risk of ASCVD. In terms of the overall effects, the effects of engaging in Baduanjin and aerobic exercise for one year were similar. However, notably, engaging in Baduanjin was better than aerobic exercise in terms of regulating HDL-C in patients with prediabetes, whereas aerobic exercise was better than engaging in Baduanjin in terms of reducing HbA1c and BP. Thus, patients should choose an exercise mode according to their specific conditions to obtain greater benefits in terms of disease prevention.

Some studies have shown that when FPG exceeds 5.6 mmol/L, the related mortality risk begins to increase^21^. Further studies have found that a higher mortality risk occurs for FPG of 6.1–6.9 mmol/L^4^. Therefore, we should actively explore effective strategies to reduce FPG. Our results showed that Baduanjin or aerobic exercise significantly alleviated the abnormal FPG of patients with prediabetes in a relatively short time (about 3 months). This benefit may be because the exercise time was scheduled 1–2 h after dinner in this study. This strategy not only takes into account the willingness of patients to participate in exercise at different times, but, more importantly, highlights the possibility that postprandial exercise can better regulate FPG. Such a pattern can be observed in the typical lifestyle habits of Chinese people, as they mainly consume rice, noodles and other carbohydrates for dinner, are busy during the day, and have leisure time after dinner; this leisure time is why they are more willing to engage in “square dance” or other exercise at this time. The research of Andrew n. Reynolds et al.^22^ shows that compared with daily walks at varying times, the incremental area under the curve (iAUC) of blood glucose is significantly reduced by daily walks after dinner, especially for those who mainly consume carbohydrates and are sedentary. Therefore, exercise after dinner is a more suitable time for Chinese patients with prediabetes^23^. In the absence of exercise, circadian rhythmsexert obvious effects on blood glucose and insulin; at night, the ability of islet β cells to secrete insulin decreases, resulting in an overnight increase in the blood glucose of people with abnormal glucose metabolism^24^. The inhibitory effect of proper exercise after dinner on the rise of blood glucose has been confirmed^25^. Exercise after dinner consumes excess glucose and energy, quickly improves insulin resistance, and reduces nocturnal blood glucose and affect FPG. But given these benefits, it is logical that increased nocturnal exercise may increase the risk of hypoglycaemia. Early hypoglycaemia may occur immediately after exercise. Meanwhile, the effect of exercise on blood glucose lasts for several hours and may even lead to nocturnal hypoglycaemia or delayed hypoglycaemia. According to Li et al.’s^26^ research, patients with T2DM who exercise for a short duration and moderate intensity after dinner can reduce blood glucose without a potential risk of delayed hypoglycaemia. However, the risk of hypoglycaemia may be increased by exercising before meals or for a long duration. In our study, we scheduled 60 min of Baduanjin exercise (low-intensity aerobic exercise) or moderate-intensity aerobic exercise after dinner, and no individuals experienced hypoglycaemic events. This lack of hypoglycaemic events may be because our exercise was arranged 1–2 h after dinner instead of late at night; our subjects were patients with prediabetes rather than T2DM, and thus their ability to regulate and tolerate blood glucose fluctuations was greater. We also found that the FPG of the participants decreased significantly after 3–6 months of the exercise intervention; subsequently, blood glucose remained stable. Therefore, we did not observe a continuous decrease in FPG. This pattern may be because 3–6 months of Baduanjin and aerobic exercise increased the ability of insulin to stimulate glucose transporter type 4 (GLUT4) in muscle cells, increased glucose transport and lipid utilization, and thus reduced blood glucose. In the meantime, to maintain the dynamic balance of blood glucose, the body also normalized the blood glucose level through breakdown of liver glycogen and gluconeogenesis, allowing the body to gradually adapt to the exercise-induced changes and restore blood glucose regulation to normal^27^.

In this study, the two exercise forms effectively reduced the blood glucose (the FPG and 2-hPG) of patients with prediabetes, contributing to normalization of blood glucose, with similar effects. Baduanjin, a low-intensity aerobic exercise, achieved effects similar to those of medium-intensity aerobic exercise, possibly because Baduanjin requires static power^28^. To practise Baduanjin, skeletal muscles must undergo centripetal, centrifugal and isometric contraction and coordinate the activity of antagonistic muscle pairs to maintain postural stability. This exercise leads to muscle fibre thickening, increases muscle strength and volume, improves muscle absorption and utilization of glucose and lipids, and thus reduces blood glucose. Notably, one participant in the BDJ group with prediabetes transitioned to T2DM by the 12th month of the intervention. We thoroughly reviewed the data of this patient and found that his blood glucose level (FPG = 6.8 mmol/L, 2-hPG = 10.5 mmol/L) at the time of registration was very close to the critical value for the diagnosis of diabetes. However, in the AT group, 4 participants had the same blood glucose level but did not transition to diabetes, possibly because compared with Baduanjin exercise, aerobic exercise significantly reduces HbA1c within three months, provides valuable time to reverse the disease progression. In contrast, Baduanjin exercise has a relatively slow effect, making it difficult to reverse the disease progression of such patients. These findings are consistent with the results of Yu et al*.*^29^ who suggested that the intervention duration of traditional Chinese exercises must exceed 6 months to obtain obvious effects. However, further research and large-scale data are needed to verify these theories.

WC is a sensitive measure of abdominal obesity that reflects the degree of fat accumulation in the abdominal cavity and viscera. WC > 85 cm is closely related to the incidence of diabetes and cardiovascular complications^30^. For every 1-cm increase in WC, the risk of CVD increased by 3.2%^31^. In this study, most participants had abdominal obesity, but after one year of Baduanjin or aerobic exercise, the WC of participants in the BDJ and AT groups decreased by 7% and 9.2%, respectively, and their weight decreased by 6.63% and 7.81%. Reducing body weight by 5–10% reduces the risk of CVD^32^. Thus, Baduanjin and aerobic exercise can effectively reduce the CVD risk of patients with prediabetes by reducing WC and weight.

Control of abdominal obesity facilitates alleviation of dyslipidaemia. In this study, after intervention, the dyslipidemia of prediabetes patients in the two exercise groups was significantly improved. Notably, HDL-C in the BDJ group was significantly higher than that in the AT group, indicating that Baduanjin exercise better regulated the HDL-C of patients with prediabetes. This result is not completely consistent with the results of a study by Mei et al.^33^. They systematically analysed a total of 14 studies on the regulation of blood lipid metabolism through practise of Baduanjin. While practising Baduanjin helped to regulate dyslipidaemiain these patients, it had no advantages compared with other exercise forms. This discrepancy may be because the intervention duration of the included studies was shorter, generally 3–6 months; in contrast, our study spanned 12 months, which was sufficient time to fully exert the benefits of Baduanjin. Effects of traditional Chinese medicine on diseases often take longer to appear than those of Western medicine; similarly, traditional Chinese medicine advocates gentle, long-term persistence to reconcile the body and mind. Baduanjin, a nondrug method of traditional Chinese medicine, may have a similar slower effect. However, this form of exercise is easier for patients as it encompasses eight simple actions. For most patients with prediabetes who are mostly sedentary, simple exercise can provide large benefits.

We found that both exercise forms significantly reduced the BP of patients with prediabetes. Aerobic exercise exhibited more advantages for BP control than Baduanjin exercise, which is why moderate-intensity aerobic exercise is recommended as a first-line intervention by national guidelines for the prevention and treatment of hypertension^34^. However, Baduanjin exercise also helped to control BP. Xiong et al.^35^ conducted a meta-analysis on the effect of Baduanjin practise on hypertension, including 1,058 participants from 14 RCTs. They found that Baduanjin exercise helped to control blood pressure, but there was little evidence that Baduanjin exercise influenced the occurrence and mortality of CVDs. A long-term RCT with a large sample size is lacking.

Prediabetes can increase the risk of CVD by 20%^36^. In our study, the control group received health guidance, but at the 12-month follow-up, their 10-year risk of ASCVD had increased by 15.50%. Thus, only simple knowledge has little effect on the blood glucose status and cardiovascular risk of patients with prediabetes, similar to how most smokers know that smoking is harmful for their health but do not take any action to change their habits. To reverse sedentary behaviour and thus prevent diabetes and its complications, practical actions are needed to combine health education with exercise intervention and attract and drive prediabetic patients and their close contacts to change their lifestyle through rich and diverse forms of exercise. In our study, compared with the control group, Baduanjin and aerobic exercise had obvious preventive and protective effects on the 10-year risk of ASCVD in patients with prediabetes. Baduanjin exercise effectively reduced the risk of ASCVD (reduced by about 33%) and achieved a similar effect as moderate-intensity aerobic exercise. The similar effects may result from similarities in the long-term practise of Baduanjin with moderate-intensity aerobic exercise, which effectively improves glucose and lipid metabolism in patients with prediabetes, regulates BP, and reduces abdominal obesity. Additionally, during our study, we found that participants in the BDJ group felt more energetic and felt a greater sense of peace after long-term practise. This change in mood may be related to Baduanjin exercise-induced improvements in depression and sleep disorders^37^. Maintenance of good mood and regular work and rest benefits endocrine regulation. Moreover, the instructor in the BDJ group provided information regarding the health-preserving benefits of traditional Chinese medicine and facilitated discussion to improve the self-awareness, which may be an additional benefit of practising Baduanjin. The combination of these factors helped to reverse disease progression in patients with prediabetes and reduce the risk of ASCVD.

## Methods

### Design and randomization

This study was a single-blind RCT. After obtaining informed consent from all participants who met the inclusion criteria, the first author anonymized the information of participants by assigning them a unique code. Participants were then randomly assigned to one of three parallel groups according to a computer-generated number sequence; the 3 groups were as follows: the Baduanjin(BDJ), aerobic training(AT) and control groups. Researchers who assessed outcomes and performed statistical analysis were blinded to the group assignments of participants. Assessments were conducted at four time points: before the intervention (baseline) and at 3rd, 6th, and 12th month of intervention.

### Setting and participants

Participants were recruited through purposive sampling. This study was conducted at three community health service centres (Baichunyuan, Zhongxin Road and Hongqi Road) in Shaoyang City, Hunan Province, southern China. We identified previously diagnosed prediabetic patients or individuals at high risk of diabetes within the community health records. Eligible patients were contacted by telephone and provided with a screening appointment at their local community health service centre. The inclusion criteria were as follows: (1) age of 40–70 years; (2) sedentary lifestyle; (3) passed the pre-exercise assessment, no movement disorders or contraindications; and (4) diagnosed withprediabetes^2^. A diagnosis of prediabetes was defined as impaired fasting glucose[IFG; fasting plasma glucose (FPG) of 6.1 to < 7.0 mmol/L], and 2-h blood glucose (2-h PG) < 7.8 mmol/L after a 75-g oral glucose tolerance test (OGTT); impaired glucose tolerance (IGT) was defined as FPG < 7.0 mmol/L, and 2-h PG of 7.8 to 11.1 mmol/L. Participants were excluded if they (1) had uncontrolled or severe acute or chronic diseases, mental illness, cancer or other immune diseases; (2) had a history of alcoholism in the past year; (3) used hypoglycaemic drugs or corticosteroids in the past or during the intervention; or (4) did not complete the study.

### Sample size

The necessary sample size was estimated as follows: $n = \frac{{\phi 2\left( {\sum\nolimits_{i = 1}^{k} {{{S_{i}^{2} } \mathord{\left/ {\vphantom {{S_{i}^{2} } k}} \right. \kern-\nulldelimiterspace} k}} } \right)}}{{\sum\nolimits_{i = 1}^{k} {{{\left( {\chi_{i} - \chi } \right)^{2} } \mathord{\left/ {\vphantom {{\left( {\chi_{i} - \chi } \right)^{2} } {\left( {k - 1} \right)}}} \right. \kern-\nulldelimiterspace} {\left( {k - 1} \right)}}} }}$. To determine significant differences among the three groups, the groups needed to include at least 25 patients. Thus, the total sample size was calculated as 75, and the dropout rate was set at 20%, resulting in a total necessary sample size of at least 94 patients.

### Intervention measures

#### Diabetes education

All participants in the three groups received diabetes health education through the community health service centres on the topics of healthy diet, exercise, diabetes self-management, and observation of disease condition before the exercise intervention. Each topic was taught once in a 1-h session, with 4 sessions in total. Instruction in this course was provided by a diabetes specialist nurse with 10 years of experience; at the end of the course, all subjects were asked to take a test to determine whether the participants demonstrated understanding of the relevant knowledge.

#### Control group

During the study, we advised participants to adhere to the principles of a healthy diet, exercise according to their own wishes or maintain their original exercise habits. A researcher supervised the process and recorded the daily exercise habits of participants by means of exercise bracelets and WeChat exercise applets; the subjects of the control group were invited to participate in the measurements and face-to-face interviews before the intervention and at the 3rd, 6th and 12th months of follow-up.

#### Pre-exercise test of the two exercise groups

A professional exercise rehabilitation doctor with more than 5 years of working experience administered the Physical Activity Readiness Questionnaire (PAR-Q) to provide a safety assessment for the preliminary participants before the exercise load test. Participants who passed the PAR-Q assessment were then presented with a bicycle ergometer for graded exercise testing over 20 min. During this period, the cardiopulmonary reserve function, reserve oxygen uptake and metabolic equivalent were evaluated, and the exercise safety of subjects was determined in combination with the above data^38^. Two weeks before the intervention, participants received additional instruction (mainly adaptive training and learning technical movements) to gradually adapt to the expected exercise intensity, which was adjusted according to the individual participants.

#### Exercise interventions

1. The AT group. In this group, according to the guidelines of the American College of Sports Medicine^39^ and referring to the exercise prescription for pre diabetes formulated by Luo et al*.*^40^, the research team and the fitness coach of the aerobic group jointly formulated the aerobic exercise program. The participants performed moderate-intensity aerobic exercise, mainly rhythmic exercises and square dances. Their heart rate (HR) was monitored every 15 s using a wireless heart rate transmitter to maintain exercise at a moderate intensity level (an intensity that causes noticeable increases in breathing and HR, 40 ~ 60% heart rate reserve, 12–13 ratings of perceived exertion^38^).
2. The BDJ group. According to the "Health Qigong—Baduanjin" standards issued by the General Administration of Exercise (GASC)^41^, one complete Baduanjin round takes 15–20 min(It was described https://v.qq.com/x/page/m0938jukfsh.html)^42^. Referring to the relevant literature^14,18,43^, we instructed the participants topractise2 ~ 3 rounds of Baduanjin in each exercise session.
3. Exercise duration and frequency of the two exercise groups. From a survey of exercise motivation, we observed that participants were most willing to participate in group training at 7–9 PM. Therefore, we decided to set the training time at 19:30–20:30 (approximately 1–2 h after dinner). Both exercise groups were allowed to warm up for 10 min beforehand. Group training lasted 40–45 min, and participants were allowed to stretch and relax for 5–10 min after exercise, for a total of 60 min. The two exercise groups were trained every other day: the AT group was trained on odd days, and the BDJ group was trained on even days. The average cumulative number of training sessions per month was 15, and the intervention lasted for a total of 12 months.

#### Quality control

The Shaoyang Exercise Association has two professional coaches with more than 5 years of training experience; these coaches were responsible for training in aerobics and Baduanjin, respectively. The coaches provided training for participants in two different venues. After the participants fully mastered the exercises, two researchers who had received advance training took charge of each group to continue to administer training and supervise the implementation of the exercise plan. The coaches provided weekly guidance to ensure that participant movements were standardized and that the desired exercise intensity was reached. Participants were excluded if their monthly attendance rate was less than 70%^44^. The 4 measurements and medical advice were all free. Sometimes, we gave some small gifts (eggs, toothpaste, towels, etc.) to encourage the participants to adhere to the treatment.

### Measurements

#### Baseline data

Before the intervention, a structured questionnaire was used to collect participant demographic and clinical characteristics, including sex, years of education, use of antihypertensive drugs, smoking status, and family history of ASCVD.WC was measured with a flexible ruler to the nearest centimetre (cm). Body weight and height were measured in a standing position by using an electronic scale to the nearest 0.1 kg or cm. Body mass index (BMI) was defined as weight (kg)/height (m)^2^. Obesity was assessed according to BMI, with the following categories: normal (18 to 24 kg/m^2^), overweight (24 to 28 kg/m^2^), and obese (≥ 28 kg/m^2^). The waist-to-height ratio (WHtR) was calculated as WC (cm)/height (cm); a WHtR > 0.5 indicated abdominal obesity.

#### Primary outcomes

1. Laboratory indicators: After 8 h of fasting overnight, body weight and WC were measured at 7:00 AM the next morning, and venous blood was collected to measure fasting plasma glucose (FPG), glycosylated haemoglobin (HbA1c), total cholesterol (TC), triglycerides (TG), high-density lipoprotein cholesterol (HDL-C), and low-density lipoprotein cholesterol (LDL-C). After fasting blood collection, the patients took 75 g of glucose orally, and their 2-hPGwas measured. Biochemical indicators were assessed by an automatic biochemical instrument (Hitachi, product type: 7600), and HbA1c was measured by a Variant II HbA1c analyser (Bio-Rad, product type: 270-2001). After sitting for 15 min, the blood pressure (BP) of participants was measured with a standard mercury sphygmomanometer.
2. Ten-year risk of ASCVD: The predicted 10-year risk of ASCVD was obtained using the Prediction for ASCVD Risk in China (China-PAR) equations, which are appropriate for the Chinese population^45–47^. Participant information was input into the web-based evaluation tool of the China-PAR model (http://www.cvdrisk.com.cn); this information included their sex, age, residential region (urban or rural), geographic region (North or South, using the Yangtze River as a boundary), WC, TC, HDL-C, treated or untreated systolic BP (SBP), diabetes (yes or no), current smoking (yes or no), and family history of ASCVD (atherosclerosis, heart attack or stroke caused by cardiovascular or cerebrovascular diseases). If the 10-year risk of ASCVD ≥ 10.0%, the risk was considered high; moderate risk was defined as 5.0%-9.9%; and low risk was defined as < 5.0%.

#### Secondary outcome

The glucose status of participants was determined according to their results on the 75-g oral glucose tolerance test after an overnight fast. The diagnostic criteria for T2DM^2^ were as follows: FPG ≥ 7.0 mmol/L and/or 2-hPG ≥ 11.1 mmol/L. Normal glucose regulation (NGR)was defined as FPG < 6.1 mmol/L and 2-hPG < 7.8 mmol/L.

### Ethical considerations

This research protocol was reviewed and approved by the Ethics Committee of Shaoyang University, and all procedures followed the recommendations of the International Ethical Guidelines for Human Biomedical Research and the Declaration of Helsinki. In addition, this study was registered with the Chinese Clinical Trials Registry (Registration number: ChiCTR1900026108, registration date: 21/09/2019). All subjects participated voluntarily and provided informed consent. The patients were informed that they were free to withdraw for any reason at any time during the study without any explanation and that the data set would remain confidential.

### Statistical Analysis

SPSS 23.0 (SPSS Inc, Chicago, IL, USA) software was used for statistical analysis. Chi square test was used for analysis of all count data, and one-way ANOVA was used to compare baseline demographic characteristics. Two-factor repeated measurement ANOVA was used to analyze each dependent variable over time (from baseline to12 months) and also analyze the group × time interaction. When the Mauchly’s sphericity test was not assumed, the test results were subjected to the results of multivariate ANOVA. (M_12_ − M_0_)/M_0_ was used to calculate the % change of the outcome measures, the M_0_ was the mean value of the baseline and the M_12_ was the mean value of the 12th month of the intervention. Mean difference (95%IC) was used to show the mean difference before and after within groups from baseline to measurements. The effect sizes of repeated measurement ANOVA was expressed by partial eta-squared (η^2^p; small ≥ 0.01, medium ≥ 0.06, large ≥ 0.14). The effect sizes of mean difference between groups was expressed by Cohen’s d (d; small ≥ 0.2, medium ≥ 0.5, large ≥ 0.8). Cox proportional hazards model was used to calculate the 10-year ASCVD risk change into low risk, and the height, age, sex, Current smoker, use of antihypertensive drugs, family history and other parameters were corrected. A statistically significant level was defined as 0.05.

## Limitations of the study

There are some limitations of this study. The sample size was relatively small, and we did not observe an effect of exercise on the 10-year risk of ASCVD in prediabetic patients aged 20–40 years. However, given the impacts of COVID-19 and the influence of environmental, social and other factors, the loss rate was slightly higher than expected. We used mobile internet technology to facilitate communication, ensure follow-up, and supervise subjects to keep these adverse factors from affecting our final analysis. Future research would benefit from increasing the sample size, performing further subgroup analysis and optimizing the study design.

## Conclusion

Our study found that Baduanjin exercise effectively reduced the risk of T2DM and its cardiovascular complications by alleviating or reducing hyperglycaemia, hyperlipidaemia, hypertension and abdominal obesity in prediabetic patients. The effects of Baduanjin exercise were similar to those of moderate-intensity aerobic exercise. We recommend that clinical health care workers and middle-aged and elderly patients who are not able to tolerate moderate aerobic exercise use this low-intensity, safe and simple exercise to prevent and control diseases.

### Clinical messages

- Long-term Baduanjin training effectively alleviated glucose and lipid metabolism abnormalities, hypertension and abdominal obesity in prediabetic patients, thereby reducing the risk of type 2 diabetes mellitus and the 10-year risk of atherosclerotic cardiovascular disease.
- Baduanjin is simple, easy to learn, safe and feasible; it is therefore recommended.

## Data Availability

The datasets generated and/or analysed during the current study are not publicly available due the research is still in progress, but are available from the corresponding author on reasonable request.

## References

[1] International Diabetes Federation. *IDF Diabetes Atlas 10th Edition*. (International Diabetes Federation, 2022).

[2] Diabetes Branch of Chinese Medical Association (2018). Guidelines for prevention and treatment of type 2 diabetes in China (2017 Edition). Chin. J. Diabetes Mellit..

[3] Li G (2008). The long-term effect of lifestyle interventions to prevent diabetes in the China Da Qing diabetes prevention study: A 20-year follow-up study. Lancet.

[4] Cai X (2020). Association between prediabetes and risk of all cause mortality and cardiovascular disease: Updated meta-analysis. BMJ.

[5] Spanakis EK, Golden SH (2013). Race/ethnic difference in diabetes and diabetic complications. Curr. Diabetes Rep..

[6] Admiraal WM (2014). Ethnic disparities in the association of impaired fasting glucose with the 10-year cumulative incidence of type 2 diabetes. Diabetes Res. Clin. Pract..

[7] Yusuf S (2020). Modifiable risk factors, cardiovascular disease, and mortality in 155 722 individuals from 21 high-income, middle-income, and low-income countries (PURE): A prospective cohort study. Lancet.

[8] American Diabetes Association (2019). 3. Prevention or delay of type 2 diabetes: Standards of medical care in diabetes—2019. Diabetes Care.

[9] Wheeler DC (2020). 2019 ESC guidelines on diabetes, pre-diabetes, and cardiovascular diseases developed in collaboration with the EASD. Eur. Heart J..

[10] Gong Q (2019). Morbidity and mortality after lifestyle intervention for people with impaired glucose tolerance: 30-year results of the Da Qing Diabetes Prevention Outcome Study. Lancet Diabetes Endocrinol..

[11] Colberg SR (2010). Exercise and type 2 diabetes. Diabetes Care.

[12] Dai X (2019). Two-year-supervised resistance training prevented diabetes incidence in people with prediabetes: A randomised control trial. Diabetes/Metab. Res. Rev..

[13] Salas-Salvadó J (2019). Effect of a lifestyle intervention program with energy-restricted Mediterranean diet and exercise on weight loss and cardiovascular risk factors: One-year results of the PREDIMED-Plus Trial. Diabetes Care.

[14] Chen M-C, Liu H-E, Huang H-Y, Chiou A-F (2012). The effect of a simple traditional exercise programme (Baduanjin exercise) on sleep quality of older adults: A randomized controlled trial. Int. J. Nurs. Stud..

[15] Wen J (2017). Baduanjin exercise for type 2 diabetes mellitus: A systematic review and meta-analysis of randomized controlled trials. Evid. Based Complement. Altern. Med..

[16] Zeng Z-P (2020). Effects of Baduanjin exercise for knee osteoarthritis: A systematic review and meta-analysis. Complement. Ther. Med..

[17] Zou L, Yeung A, Quan X, Boyden S, Wang H (2018). A systematic review and meta-analysis of mindfulness-based (Baduanjin) exercise for alleviating musculoskeletal pain and improving sleep quality in people with chronic diseases. Int. J. Environ. Res. Public Health.

[18] Chen X (2020). Intensity level and cardiorespiratory responses to Baduanjin exercise in patients with chronic heart failure. ESC Heart Fail..

[19] Zhou J (2020). Characteristic of clinical studies on Baduanjin during 2000–2019: A comprehensive review. Evid. Based Complement. Altern. Med..

[20] Ma Q, Li H, Gao Y, Zou Y (2021). Effects of Baduanjin on glucose and lipid metabolism in diabetic patients. Medicine.

[21] Seshasai SRK (2011). Diabetes mellitus, fasting glucose, and risk of cause-specific death. N. Engl. J. Med..

[22] Reynolds AN, Mann JI, Williams S, Venn BJ (2016). Advice to walk after meals is more effective for lowering postprandial glycaemia in type 2 diabetes mellitus than advice that does not specify timing: A randomised crossover study. Diabetologia.

[23] Li J, Wang L, Chen F, Xia D, Miao L (2018). Switching from glargine+insulin aspart to glargine+insulin aspart 30 before breakfast combined with exercise after dinner and dividing meals for the treatment of type 2 diabetes patients with poor glucose control – a prospective cohort study. BMC Endocr. Disord..

[24] Morris CJ (2015). Endogenous circadian system and circadian misalignment impact glucose tolerance via separate mechanisms in humans. Proc. Natl. Acad. Sci. U.S.A..

[25] Erickson ML, Little JP, Gay JL, McCully KK, Jenkins NT (2017). Effects of postmeal exercise on postprandial glucose excursions in people with type 2 diabetes treated with add-on hypoglycemic agents. Diabetes Res. Clin. Pract..

[26] Li Z (2018). Twenty minute moderate-intensity post-dinner exercise reduces the postprandial glucose response in Chinese patients with type 2 diabetes. Med. Sci. Monit..

[27] Evans PL, McMillin SL, Weyrauch LA, Witczak CA (2019). Regulation of skeletal muscle glucose transport and glucose metabolism by exercise training. Nutrients.

[28] Xiong G, Wang A, Ye H (2019). Establishment and application of Ba Duan Jin exercise prescription in obese patients with type 2 diabetes. Chin. J. Nurs. Educ..

[29] Yu D-D (2020). Effects of traditional Chinese exercises on blood glucose and hemoglobin A1c levels in patients with prediabetes: A systematic review and meta-analysis. J. Integr. Med..

[30] Ross R (2020). Waist circumference as a vital sign in clinical practice: A consensus statement from the IAS and ICCR working group on visceral obesity. Nat. Rev. Endocrinol..

[31] Tian J (2017). Contribution of birth weight and adult waist circumference to cardiovascular disease risk in a longitudinal study. Sci. Rep..

[32] Lavie CJ, Ozemek C, Carbone S, Katzmarzyk PT, Blair SN (2019). Sedentary behavior, exercise, and cardiovascular health. Circ. Res..

[33] Mei L, Chen Q, Ge L, Zheng G, Chen J (2012). Systematic review of Chinese traditional exercise baduanjin modulating the blood lipid metabolism. Evid. Based Complement. Altern. Med..

[34] Whelton PK (2018). 2017 ACC/AHA/AAPA/ABC/ACPM/AGS/APhA/ASH/ASPC/NMA/PCNA guideline for the prevention, detection, evaluation, and management of high blood pressure in adults: A report of the American College of Cardiology/American Heart Association task force on clinical practice guidelines. Hypertension.

[35] Xiong X, Wang P, Li S, Zhang Y, Li X (2015). Effect of Baduanjin exercise for hypertension: A systematic review and meta-analysis of randomized controlled trials. Maturitas.

[36] Beulens JWJ (2019). Risk and management of pre-diabetes. Eur. J. Prev. Cardiol..

[37] Cheung DST (2021). Assessing the effect of a mind-body exercise, qigong Baduanjin, on sleep disturbance among women experiencing intimate partner violence and possible mediating factors: A randomized-controlled trial. J. Clin. Sleep Med..

[38] Riebe D (2015). Updating ACSM’s recommendations for exercise preparticipation health screening. Med. Sci. Sports Exerc..

[39] American College of Sports Medicine. *ACSM’s Guidelines for Exercise Testing and Prescription 9th Ed*. (2014). 10.1249/JSR.0b013e31829a68cf 23851406

[40] Xijuan L (2014). Exercise prescription for pre-diabetes: Design and implementation. J. Beijing Sport Univ..

[41] Health Qigong Management Center of General Administration of Aports of China. *Health Qigong Baduanjin*, <https://www.sport.gov.cn/qgzx/n5407/c838019/content.html> (2017).

[42] Traditional Chinese Medicine Bureau of Guangdong Province. *Instrctions for Baduanjin*, <https://v.qq.com/x/page/m0938jukfsh.html> (2020).

[43] Ren, J. *Clinical Observation Study on Intervention of Fitness Qigong Baduanjin in Patients with Pre Diabetes, Master*. (Nanjing University of traditional Chinese Medicine, 2014).

[44] Dai X (2019). Two-year-supervised resistance training prevented diabetes incidence in people with prediabetes: A randomised control trial. Diabetes/Metab. Res. Rev..

[45] Joint Task Force for Guideline on the Assessment and Management of Cardiovascular Risk in China. Guideline on the assessment and management of cardiovascular risk in China. *Chin. Circ. J.* **34**, 4–28, doi:10.3969/j.issn.1000-3614.2019.01.002 (2019).

[46] Yang X (2016). Predicting the 10-year risks of atherosclerotic cardiovascular disease in Chinese population. Circulation.

[47] Yang XL (2016). Risk stratification of atherosclerotic cardiovascular disease in Chinese adults. Chronic Dis. Transl. Med..

