# Author Correction: A randomized controlled trial of Baduanjin exercise to reduce the risk of atherosclerotic cardiovascular disease in patients with prediabetes

**DOI:** 10.1038/s41598-023-28607-y

**Published:** 2023-01-24

**Authors:** Xiaojun Ma, Manlin Li, Lin Liu, Fenfang Lei, Liduo Wang, Wenyan Xiao, Yingzi Tan, Binghua He, Sijie Ruan

**Affiliations:** 1 School of Nursing, Shaoyang University, Hunan Province, Shaoyang, 422000 China; 2 Graduate School, Adamson University, 0900 Manila, Philippines; 3 School of Nursing, Yangzhou University, Yangzhou, 225009 Jiangsu Province China; 4 Department of Anesthesiology, Central Hospital of Shaoyang, Hunan Province, Shaoyang City, 422000 China

Correction to: *Scientific Reports* 10.1038/s41598-022-22896-5, published online 11 November 2022

In the original version of this Article Lin Liu was incorrectly affiliated with ‘School of Nursing, Shaoyang University, Shaoyang, 422000, Hunan Province, China’. The correct affiliation is listed below.

School of Nursing, Yangzhou University, Yangzhou, 225009, Jiangsu Province, China.

In addition, Fenfang Lei was incorrectly affiliated with ‘Graduate School, Guangxi Medical University, Nanning, 530022, Guangxi Zhuang Autonomous Region, China’ and ‘Department of Anesthesiology, Central Hospital of Shaoyang, Shaoyang City, 422000, Hunan Province, China’. The correct affiliation is listed below.

School of Nursing, Shaoyang University, Shaoyang, 422000, Hunan Province, China.

The original Article has been corrected.

